# Pitahaya (*Hylocereus ocamponis*)-Peel and -Flesh Flour Obtained from Fruit Co-Products—Assessment of Chemical, Techno-Functional and In Vitro Antioxidant Properties

**DOI:** 10.3390/molecules29102241

**Published:** 2024-05-10

**Authors:** Verónica Reyes-García, Carmen Botella-Martínez, Naida Juárez-Trujillo, Nuria Muñoz-Tébar, Manuel Viuda-Martos

**Affiliations:** 1 Tecnológico Nacional de México/I.T. del Altiplano de Tlaxcala, Carr. Federal San Martin-Tlaxcala Km 7.5, San Diego Xocoyucan 90122, TL, Mexico; veronica.rg@altiplano.tecnm.mx; 2 IPOA Research Group, Instituto de Investigación e Innovación Agroalimentaria y Agroambiental, Universidad Miguel Hernández (CIAGRO-UMH), 03312 Orihuela, Alicante, Spain; c.botella@umh.es (C.B.-M.); nmunoz@umh.es (N.M.-T.); 3 Centro de Investigación y Desarrollo en Alimentos, Universidad Veracruzana, Av. Dr. Luis Castelazo Ayala s/n Industrial animas CP, Xalapa 91192, VC, Mexico; naijua1993@gmail.com

**Keywords:** dragon fruit, pitaya, bioactive compounds, polyphenolic compounds, betalains

## Abstract

The aim of this work was to assess the chemical composition and physico-chemical, techno-functional, and in vitro antioxidant properties of flours obtained from the peel and flesh of pitahaya (*Hylocereus ocamponis*) to determine their potential for use as ingredients for food enrichment. The chemical composition, including total betalains, mineral content, and polyphenolic profile, was determined. The techno-functional properties (water holding, oil holding, and swelling capacities) were also evaluated. For the antioxidant capacity, four different methodologies, namely ferrous ion-chelating ability assay, ferric-reducing antioxidant power assay; 1,1-Diphenyl-2-picrylhydrazyl radical scavenging ability assay, and 2,2′-azino-bis(3-ethylbenzothiazoline-6-sulfonic acid) radical assay, were used. Pitahaya-peel flour had higher values for protein (6.72 g/100 g), ash (11.63 g/100 g), and dietary fiber 56.56 g/100 g) than pitahaya-flesh flour, with values of 6.06, 3.63, and 8.22 g/100 g for protein, ash, and dietary fiber, respectively. In the same way, pitahaya peel showed a higher content of minerals, betalains, and polyphenolic compounds than pitahaya-flesh flour, with potassium (4.43 g/100 g), catechin (25.85 mg/g), quercetin-3-rhamnoside (11.66 mg/g) and myricetrin (12.10 mg/g) as principal compounds found in the peel. Again, pitahaya-peel flour showed better techno-functional and antioxidant properties than pitahaya-flesh flour. The results obtained suggest that the flours obtained from the peel and pulp of pitahaya (*H. ocamponis*) constitute a potential material to be utilized as an ingredient in the food industry due to the high content of bioactive compounds such as betalains, phenolic acids, and flavonoids, with notable antioxidant capacity.

## 1. Introduction

The fruit and vegetable processing industries are characterized by generating large volumes of waste that, due to their composition, are highly perishable and susceptible to generating serious environmental problems. These wastes can be considered co-products since in their composition it is still possible to find compounds with high added value such as dietary fiber, pigments, mineral vitamins, and polyphenolic compounds, among others [1]. For this reason, in recent years, there has been a growing interest, on the part of the agri-food industry, in the valorization of these co-products to obtain new ingredients that can be used in new production processes. On the other hand, in a large number of European countries, the consumption of exotic fruits, both fresh and industrialized, including durian, moringa, lychee, noni, and rambutan, among others, has increased significantly [2] due to the flavors, aromas, and textures that these fruits present. In the same way, these fruits and their co-products may help maintain health and prevent the development of several diseases [3].

Among the exotic fruits that have aroused the interest of European consumers is the pitaya (pitahaya, or dragon fruit) (*Hylocereus* spp.), which belongs to the Cactaceae family. Pitahaya is a fruit widely cultivated and consumed in Latin America and Southeast Asia, both fresh and industrialized in the form of juice, wine, ice creams, jam, or jellies. This fruit is very attractive due to its shape and the colors of both the pulp and the skin. From a commercial point of view, three varieties are widely distributed around the world. These varieties are *Hylocereus megalanthus*, with yellow skin and white pulp; *Hylocereus undatus*, with red skin and white pulp; and *Hylocereus polyrhizus*, with red peel and red pulp [4]. A different type of pitahaya, known as *Hylocereus ocamponis*, primarily inhabits the central region of Mexico, across from Nayarit to Guatemala, and it is not as commonly found in commercial markets globally [5]. The distinctive feature of this species is its fruits, which exhibit a strong pink color both in their pulp and in the peel.

The commercial pitahaya species (*H. megalanthus*, *H. undatus*, and *H. polyrhizus*) have been deeply studied, and several phytochemicals have been found in their composition, with betalains, dietary fiber, and polyphenolic compounds attracting the most interest and being found in higher concentrations [6,7,8]. These bioactive compounds of pitahaya have demonstrated several beneficial health effects such as anti-diabetic, anti-inflammatory, antioxidant, anti-cancer, and antimicrobial effects, among others [9].

The agri-food industry, as well as the scientific community, is no stranger to the enormous possibilities that pitahaya presents, and as a consequence, numerous studies have been directed at the application of flours, extracts, etc., obtained from the valorization of pitahaya co-products in the development of different foods such as dairy products, meat products, and bakery products [10,11,12]. This research aimed to assess the chemical composition and physico-chemical, techno-functional, and in vitro antioxidant properties of flours obtained from the peel and pulp of pitahaya (*Hylocereus ocamponis*) to determine the potential use of the co-products obtained from this fruit as ingredients for food enrichment.

## 2. Results

### 2.1. Chemical Composition

Table 1 shows the chemical composition of flours obtained from edible and non-edible portions of pitahaya (*H. ocamponis*) fruit. Pitahaya-peel flour had higher values (*p* < 0.05) for protein, ash, and dietary fiber than pitahaya-flesh flour; for the fat content, no statistical differences (*p* > 0.05) were obtained between both flours.

In reference to pitahaya-peel flour, in the scientific literature, there is a great variability in the chemical composition, which depends on the species of pitahaya analyzed. In this sense, Moreira Morais et al. [13] report that the fat and protein content (1.56 and 5.45 g/100 g, respectively) of the pitahaya (*Hylocereus costaricensis*)-peel flour was lower than that obtained in this research work but had a higher content of fiber and ash (60.21 and 19.23 g/100 g). Similarly, Utpott et al. [14] noted that the flour obtained from pitahaya (*Hylocereus polyrhizus*) peel had a protein and lipid content similar to that found in this research but the content of ash and dietary fiber was higher (17.56 and 65.59 g/100 g). Madane et al. [10] stated that the values of total dietary fiber and fat (56.91 and 2.34 g/100 g, respectively) of the pitahaya flour (*Hylocereus undatus*) were similar to those obtained in this study. However, this flour had a higher content of proteins (10.36 g/100 g). On the other hand, for pitahaya-flesh flour, the values obtained for chemical composition were similar, except for total dietary fiber, to those reported by Hernandez-Ramos et al. [6] for *H. ocamponis* flesh. However, Liaotrakoon [15] reported that *H. polyrhizus* flesh showed lower protein and fat content but a higher content of ash and dietary fiber than was obtained in this work.

Table 1 shows the pectin content, and again, the pitahaya-peel flour showed higher values (*p* < 0.05) than pitahaya-flesh flour. The pectin content in *H. ocamponis* peel was higher than reported by Liu et al. [16] in *H. undutus* peels (17.13%) and Rahmati et al. [17], who stated that the pectin content of *H. polyrhizus* peels varied between 11.8 and 18.5% depending on extraction conditions. This high total dietary fiber content and high pectin content make pitahaya-peel flour a very interesting ingredient for the development of functional foods enriched and/or fortified with fiber due to the positive health effects of fiber intake, including a protective effect against cardiovascular disease, improvement of gastrointestinal health, weight management, reduction of the risk of suffering different types of cancer, etc. [18]. As regards mineral content (Table 1), the principal elements found in both *H. ocamponis*-peel and -flesh flours were potassium, calcium, and phosphorus, with statistical differences (*p* < 0.05) between flours. Magnesium, copper, sodium, zinc, and iron were also determined with values, for all elements, lower than 2 mg/100 g. The values obtained agree with those reported by Moreira Morais et al. [13] and Hernandez-Ramos et al. [6], who stated that potassium, calcium, and phosphorus were the main elements found in peels of *H. costaricensis* and *H. ocamponis*. It is important to highlight this high potassium content. Potassium exerts multiple effects on the organism that are included in the health claims of European regulations [19].

### 2.2. Physico-Chemical Properties

The physico-chemical properties (pH, water activity, and color coordinates) of flours obtained from edible and non-edible portions of pitahaya (*H. ocamponis*) fruit are shown in Table 2. As regards pH values, the flours obtained from pitahaya peel showed lower values (*p* < 0.05) than the flours obtained from the flesh. In the same way, the water activity of pitahaya-peel flour was also lower (*p* < 0.05) than the values obtained for pitahaya-flesh flour.

These parameters are hugely related to product deterioration; thus, the low water activity and pH values of both pitahaya-peel and -flesh flours indicate that the degree of degradation caused by several factors, including enzymatic or non-enzymatic reactions, as well as by bacterial or fungal action, is minimal [20].

A very important parameter when trying to use co-products as ingredients in the development of new foods is color. Significant modifications in this parameter when incorporated into a food matrix can lead to rejection by the consumer. Therefore, knowing the value of this property is of utmost importance. The values of the color parameters are shown in Table 2. For lightness (L*) and yellowness (b*), pitahaya-peel flour showed higher values (*p* < 0.05) than pitahaya-flesh flour. However, for redness (a*), pitahaya-flesh flour had the highest values (*p* < 0.05). In the scientific literature, there are no studies about the color of flours obtained from pitahaya (*H. ocamponis*) flesh or peel. Nevertheless, in the case of flours obtained from peel, it is possible to find several works where the color coordinates of other *Hylocereus* species were determined. Thus, the values obtained in this work for pitahaya-peel flour were similar to those reported by Mai et al. [21], who stated that *H. undatus*-peel powder had L*, a*, and b* values of 50.47, 39.53, and −4.62, respectively. Utpott et al. [14] stated that the flour obtained from *H. polyrhizus* flour had values for the L*, a*, and b* coordinates of 30.22, 5.56, and −0.44, respectively. This large variability may be due to the *Hylocereus* species, the content and type of colored bioactive compounds, the particle size, the moisture content, and the state of ripeness, among other aspects.

### 2.3. Techno-Functional Properties

To determine the techno-functional properties of flours obtained from pitahaya (*H. ocamponis*)-flesh and -peel co-products, the water-holding capacity (WHC), oil-holding capacity (OHC), and swelling capacity (SWC) were assessed. These hydration properties are shown in Table 3. The results obtained were expected since it is a plant material with a high content of total dietary fiber. Flours from vegetable co-products usually have good hydration properties.

In the three parameters analyzed, the flours obtained from the peel showed higher values (*p* < 0.05) than those obtained from the flesh. Again, to our knowledge, there are no studies on the techno-functional properties of flours obtained from pitahaya flesh or peel obtained from *H. ocamponis*. However, in the case of flours obtained from peel, it is possible to find several works of other *Hylocereus* species. Thus, the values of WHC, OHC, and SWC of pitahaya-peel flour obtained in this research were higher than those obtained by Chia and Chong [22], who reported that the flour obtained from *H. polyrhizus* peel had a WHC, OHC, and SWC of 2.52 g water/g sample, 3.56 g oil/g sample, and 6.23 mL/g, respectively, and those reported by Chumroenvidhayakul et al. [23], who stated that the WHC, OHC, and SWC values of dragon fruit peel were 9.44 g water/g sample, 1.93 g oil/g sample, and 4.60 mL/g, respectively. Nevertheless, Corimayhua-Silva et al. [24] mentioned that the values of WHC and SWC of flours obtained from *Hylocereus hybridum* and *Hylocereus undatus* were higher than those obtained in this work, with values of 31.9 g water/g sample and 46.9 mL/g for *H. hibridum* and 17.3 and 41.5 for *H. undatus*. However, these flours had lower values of OHC. This variability of results shown in the scientific literature could be explained by the fact that these hydration properties depend on different factors such as particle size, dietary fiber content, fiber composition, temperature, pH, and porosity, among others [25]. For Bala et al. [26], the SWC depends on numerous factors including the proportions of the particles, the variety type, several processing techniques, and effectiveness. Normally, the WHC of flour or powder obtained from fruits or vegetables is closely related to the presence of hydrophilic components, the concentration of insoluble fiber, and the particle size, whilst the OHC was associated with the chemical and physical structure of the polysaccharides, the protein conformation, and the surface hydrophobicity, as mentioned Martinez et al. [27].

### 2.4. Antioxidant Properties

When extracts containing a complex mix of bioactive compounds are obtained from plant material and used to determine antioxidant activity, different mechanisms of action can occur. For this reason, it is important to use different antioxidant methods to obtain a broad view of the antioxidant activity of a plant extract [28]. In this work, four different methodologies with different action mechanisms, namely ferrous ion-chelating ability assay (FIC), ferric-reducing antioxidant power (FRAP) assay; 1,1-Diphenyl-2-picrylhydrazyl radical scavenging ability (DPPH) assay; and 2,2′-azino-bis(3-ethylbenzothiazoline-6-sulfonic acid) radical (ABTS) assay, were used. The results of the antioxidant capacity of both pitahaya flours, using the different methodologies, are summarized in Table 4.

When the antioxidant activity was analyzed by measuring the capacity to reduce ferric ions (FRAP assay), the pitahaya-skin meal showed higher values (*p* < 0.05) than the meal obtained from the pulp. The same behavior was observed when the antioxidant activity was measured by the free radical scavenging capacity (ABTS assay): pitahaya-peel flour showed higher values (*p* < 0.05) than pitahaya-flesh flour. In the case of antioxidant activity measured with DPPH, different results were obtained. The flour obtained from pitahaya flesh showed higher values (*p* < 0.05) than those obtained from the peel flour. It is important to note that these two methods employ the same mechanism of action, but the values obtained in the ABTS method are higher than those determined with the DPPH method. This may be due to the fact that in the ABTS method, the hydrophilic and lipophilic compounds exert their action, whereas this is not the case in the DPPH method, which shows more affinity for hydrophilic compounds [29]. When the ability of the pitahaya flours to chelate metals was analyzed using the FIC method (Table 4), no significant differences (*p* < 0.05) were found between the two samples. The values obtained agree with those found in the literature, which report that pitahaya peel has a strong antioxidant capacity compared to the flesh [7,30]. This fact could be explained by the fact that in the peel, the concentration of bioactive compounds with potential antioxidant properties, including phenolic acids, flavonoids, and total betalains, is higher than that found in the flesh portion [7,31]. In reference to the antioxidant activity of pitahaya-flesh flour, the values obtained in this study were lower than those reported by Hernandez-Ramos et al. [6], who stated that ABTS and FRAP values of *H. ocamponis* flesh were 20.09 and 11.64 mM Trolox Equivalent/g, respectively. In a similar study, Uslu and Özcan [32] found that the antioxidant capacity of pitahaya flesh (*H. undatus*) measured with DPPH assay was 24 mM/g. Concerning the antioxidant capacity of peel extracts, Bassey et al. [33] reported that the extracts obtained from the peel of red pitahaya *var*. red crystal and dried using a microwave had a lower antioxidant capacity (measured with ABTS (3.5 µM /g) and FRAP (20 µM TE/g)) than that obtained in this work. Similarly, Mai et al. [21] also reported that the antioxidant capacity of *H. undatus*-peel powder assessed with DPPH (2.12 mM/100 g) and FRAP assays (2.7 mM/100 g) had lower values. However, Hernandez-Ramos et al. [6] reported that the antioxidant capacity of *H. ocamponis* peel, measured with ABTS and FRAP methods, with values of 8.17 and 3.65 mMol TE/g, respectively, were higher than that obtained in this study. As mentioned above, this antioxidant activity was related to the high content of betalains and polyphenolic compounds (mainly flavonoids).

### 2.5. Bioactive Compounds

**Figure 1:**
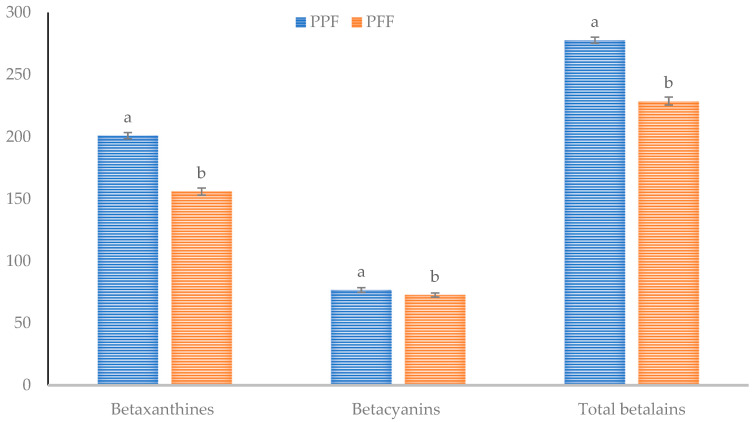
Total betalain, betacyanin, and betaxanthine content found in flours obtained from edible and non-edible portions of pitahaya (*Hylocereus ocamponis*) fruit. PPF: pitahaya-peel flour; PFF: pitahaya-flesh flour. Values for betaxanthines were expressed as mg equivalents of indicaxanthin per 100 g of sample; values for betacynins were expressed as mg equivalents of betanin per 100 g of sample. For each compound, histograms with the same letter indicate that no statistical differences were found (*p* > 0.05) according to Tukey’s HSD post hoc test.

Betalains, composed of betacyanins and betaxanthines, are natural red and yellow color pigments abundant in the red flesh and red peel of pitahaya. They have several biological effects, including antioxidant and anti-cancer properties, anti-lipidemic effects, hepatoprotective effects, and neuroprotective effects, among others [34]. The content of betacyanins and betaxanthines found in flours obtained from edible and non-edible portions of pitahaya (*H. ocamponis*) fruit is shown in Figure 1.

Pitahaya-peel flour showed a higher content of betaxanthines, betacyanins, and total betalains (*p* < 0.05) than pitahaya-flesh flour. These results agree with Suh et al. [35], who suggested that the betacyanin and betaxanthin content was higher in the peel than in the pulp. In reference to pitahaya-flesh flour, the values obtained in this study were higher than those reported by Rodrigues Vieira et al. [36], who mentioned that pitaya (*H. polyrhizus*)-pulp extracts had a betaxanthine, betacyanin, and total betalain content of 56, 110, and 156 mg/100 g, respectively. Similarly, Cheok et al. [37] stated that the betaxanthine, betacyanin, and total betalain content of the freeze-dried pulp of red dragon fruit (*H. polyrhizus*) was 21, 108, and 139 mg/100 g, respectively. As regards pitahaya peel, the values obtained were higher than those reported by Hernandez-Ramos et al. [6], who found that the betaxanthine, betacyanin, and total betalain content of *H. ocamponis*-peel flours was 3.55, 9.65, and 13.21 mg/100 g fresh weight, while in *H. undatus*-peel flours, the values for betaxanthines, betacyanins, and total betalains were 5.17, 14.66 and 19.83 mg/100 g fresh weight.

Polyphenolic compounds, present in fruits and their co-products, constitute a very important source of bioactive compounds with multiple functional properties; among these, their antioxidant activity can be highlighted. Table 5 shows the polyphenolic profile of flours obtained from edible and non-edible portions of pitahaya (*H. ocamponis*) fruit. Pitahaya-peel flour had a higher content of phenolic acids, flavan-3-ol, flavanols, and anthocyanidins (*p* < 0.05) than pitahaya-flesh flour. On the other side, the highest anthocyanin content was found in pitahaya-flesh flour. As regards phenolic acids, pitahaya-peel flour showed four phenolic acids, with 4-*O*-caffeoylquinic acid (0.97 mg/g flour) and 3-*O*-caffeoylquinic acid (0.28 mg/g flour) being in higher (*p* < 0.05) concentration, while in pitahaya-flesh flour, five phenolic acids were detected. In this case, caffeic acid (0.27 mg/ g flour) was the one that showed the highest (*p* < 0.05) concentration. In reference to the group of flavan-3-ols, only one was detected in both pitahaya flour samples. However, it is important to highlight that this compound, catechin, was found in the highest concentration of all the polyphenolic compounds detected, with values of 25.85 and 5.32 mg/g samples for pitahaya-peel flour and pitahaya-flesh flour, respectively.

For the flavonols group (Table 5), in pitahaya-peel flour, five compounds were identified, with glycosylated quercetins being the main group of components. The compounds, belonging to the flavonols group, found in the highest (*p* < 0.05) concentration were quercetin 3-rhamnoside and myricetrin, with values of 11.66 and 12.10 mg/g. For pitahaya-flesh flour, only three flavonols were detected with values lower than 0.10 mg/g. In the case of anthocyanins (Table 5), four compounds were detected in pitahaya-flesh flour (cyanidin-3-glucoside, cyanidin-3-rutinoside, delphinidin-3-glucoside, and delphinidin-3-rutinoside), whereas in pitahaya-peel flour, two compounds were identified (cyanidin-3-glucoside and delphinidin-3-glucoside). In the last group of flavonoids, the anthocyanidins (the aglycon form of anthocyanins), cyanidin and delphinidin were detected only in pitahaya-peel flour (Table 5). The results obtained in this work are opposite to those reported in the scientific literature. Thus, Tang et al. [38] analyzed the polyphenolic profile of pitahaya (*H. polyrhizus*) red peel. These authors reported that the main phenolic acids found in were chlorogenic and ferulic acids, while the principal flavonoids were quercetin and nicotiflorin. Sen and Baruah [39] analyzed the polyphenolic profile of pitahaya peel of *H. udantus* and *H. cosraricensis*. These authors informed that syringic acid and ferulic acid were the dominant phenolic acids in the peel of *H. udantus* and *H. cosraricensis,* respectively, while the main flavonoids found in both samples were ellagic acid and quercetin. In a similar study, Paśko et al. [40] stated that the main flavonoids found in pitahaya (*H. costaricensis*) flesh were myricetin and rutin. In the same way, Rodrigues Vieira et al. [36] mentioned that the main phenolic acids and flavonoids found in pitaya (*H. polyrhizus*)-pulp extracts were chlorogenic acid, rutin, and procyanidin A2. This great variability may be explained due to the concentration and type of polyphenolic compounds in fruits and their co-products depending on many factors, including the degree of ripeness of the fruit, the variety within the same species, the harvesting season, cultivation technique, and environmental factors such as the height of the growing area, climate, type of soil, among others [41].

## 3. Materials and Methods

### 3.1. Obtaining Pitahaya-Peel and -Flesh Flour

**Figure 2:**
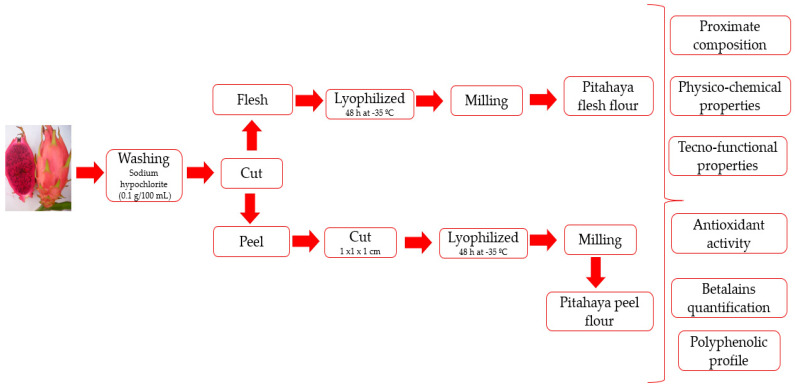
Flowchart of process to obtain flours from edible and non-edible portions of pitahaya (*Hylocereus ocamponis*) fruit, and the analytical determinations.

The pitahaya (*Heliocerus ocamponis*) fruits were obtained from a local farm in Santa Ana Teloxtoc, Tehuacán Puebla (Mexico). The fruits were transported to the Polytechnic Institute of Tlaxcala, where they were sanitized by immersion in a sodium hypochlorite solution (0.1 g/100 mL) for 15 min and washed in distilled water to eliminate chlorine residues. After that, the fruits were cut transversely, and the flesh was extracted and lyophilized in a Labconco freeze-dryer mod 121066614E (Kansas City, MO, USA) for 48 h at −35 °C. The peels were cut into pieces of 1 × 1 cm and lyophilized in a freeze-dryer for 24 h at −35 °C. The dry flesh and peels were ground separately using a mill (Cecotec, Barcelona, Spain) to obtain flour. The two fractions obtained were pitahaya-peel flour (PPF) and pitahaya-flesh flour (PFF). Both flours were vacuum-packed and stored frozen until further use. Figure 2 shows the process of obtaining the pitahaya-peel flour (PPF) and pitahaya-flesh flour, as well as the analytical determinations.

### 3.2. Proximate Composition

The chemical compositions (ash, protein, fat, and total dietary fiber) of pitahaya-peel flour and pitahaya-flesh flour were determined following the Association of Official Analytical Chemistry methods [42]. All results were expressed as g/100 g dry weight (d.w.). The pectin content was determined following the methodologies proposed by Chen et al. [43]. The mineral content was assessed by means of inductively coupled plasma–mass spectrometry (ICP–MS) using a Shimadzu MS-2030 (Shimadzu, Kyoto, Japan). The ICP–MS worked with the following specifications: carrier gas 700 mL/min; plasma gas 9000 mL/min; auxiliary gas 1100 mL/min; radio frequency 1200 W; and energy filter 7.0 V. The results are expressed as mg/100 g d.w.

### 3.3. Physico-Chemical Properties

The water activity (Aw) was analyzed with a NOVASINA TH-200 hygrometer (Novasina; Lachen, Switzerland) at 25 °C. The pH was assessed in an aqueous solution obtained from mixing 1 g of each sample with 10 mL of deionized water for 15 min, using a pH-meter pH/Ion 510 (Crison, Barcelona, Spain). The instrumental color was evaluated using a spectrophotometer CM-700 (Minolta Camera Co., Osaka, Japan). Color data (nine measurements were carried out for each flour in triplicate) were recorded as CIE L*, a*, and b* coordinates, indicating lightness, green-red, and blue-yellow, respectively.

### 3.4. Techno-Functional Properties

The water-holding capacity (WHC), oil-holding capacity (OHC), and swelling capacity (SWC) were determined according to the methodology proposed by Zhang et al. [44]. The results are presented as g of water held per g of flour, g of oil held per g of flour, and ml per g of flour, respectively.

### 3.5. Bioactive Compounds

#### 3.5.1. Extraction of Bioactive Compounds

For the extraction of betalains (betacyanins + betaxanthines) and polyphenolic compounds, pitahaya-peel or pitahaya-flesh flour were subjected to a two-stage extraction process. In the first step, 1 g of PPF or PFF was extracted by shaking at room temperature for 180 min with 10 mL of an aqueous-methanol solution (2:8 *v*/*v*). Then, the samples were centrifugated at 3500× *g* for 8 min at 4 °C, and the supernatant was collected. In the second step, the pellets were extracted by shaking at room temperature for 180 min with 10 mL of aqueous acetone (3:7 *v*/*v*). Again, the samples were centrifuged with the same conditions, and the supernatant was collected. Both supernatants were mixed and evaporated (until dry) using a rotary evaporator under reduced pressure (<100 mbar) at 40 °C. To determine the total betalain (betacyanins + betaxanthines) values and the antioxidant activity, 8 mL of methanol was added to the dried extract, and it was shaken in a vortex for 4 min. Finally, the solution was passed through a 0.45 μm filter and stored at −20 °C. In reference to the polyphenolic profile and due to the sugar content, the dried extracts of PPF or PFF were dissolved in 10 mL water and loaded onto a C-18 Sep-Pak cartridge before being eluted with 3 mL acidified methanol (0.1 g/L formic acid). The extracts obtained were maintained at −40 °C until high-performance liquid chromatography analysis.

#### 3.5.2. Antioxidant Activity

To analyze the antioxidant properties of both pitahaya-peel and pitahaya-flesh flours, four different methodologies were used. The 2,2′-azino-bis(3-ethylbenzothiazoline-6-sulfonic acid) radical (ABTS) assay was used following the procedure described by Re et al. [45]. The results were expressed in mM Trolox equivalents (TE)/g dry weight (dw). The 1,1-Diphenyl-2-picrylhydrazyl radical scavenging ability (DPPH) assay was carried out following the methodology described by Brand-Williams et al. [46]. Results were expressed in mM TE/g sample (d.w.). The ferric-reducing antioxidant power (FRAP) assay was conducted according to Yang et al. [47]. The results are expressed in mM TE/g sample (d.w.). Finally, the ferrous ion-chelating capacity assay (FIC assay) was measured according to the method reported by Mahdavi et al. [48]. The results were expressed as mg EDTA Equivalents/g flour (d.w.).

#### 3.5.3. Betalain Quantification

The content of betacyanins and betaxanthines was determined according to the method described by Wu et al. [49] using Equations (1) and (2).

(1) $$Betacyanins\;\left(\frac{mg}{100\;g}\right)=\frac{Abtc\times DF\times MWb\times V}{\varepsilon b\times L\times W}$$

(2) $$Betaxanthines\;\left(\frac{mg}{100\;g}\right)=\frac{Abtx\times DF\times MWi\times V}{\varepsilon i\times L\times W}$$

where Abtc = absorbance at 538 nm (betacyanins); Abtx = absorbance at 483 nm (betaxanthines); DF = dilution factor; MWb = molecular weight (550 g/mol for betanin); MWi = molecular weight (308 g/mol for indicaxanthin); V = gauging volume (mL); εb = molar extinction coefficient of betanin (60,000 L/mol cm); εi = molar extinction coefficient of indicaxanthin (48,000 L/mol cm); W = weight of the sample (g); and L = cell length (1 cm). The total betalains was calculated as the sum of betacyanins and betaxanthines and was expressed as mg/100 g.

#### 3.5.4. High-Performance Liquid Chromatography Analysis

The analysis of polyphenolic compounds was carried out using high-performance liquid chromatography following the methodology described by Genskowsky et al. [50] with some modifications. The extracts (20 µL) were injected into HPLC Hewlett-Packard 1200 equipped with a UV–Vis Diode Array Detector (Hewlett-Packard, Waldbronn, Germany) and a C18 Teknokroma Mediterranean sea18, (25 × 0.4 cm, 5 μm particle size) column. The chromatograms were recorded at 280, 325, 360, and 520 nm. Phenolic compounds were analyzed in standard and sample solutions using a gradient elution at 1 mL/min with the following gradient program: start with 95% A, then 75% A at 20 min, 50% A at 40 min, 20% A at 50 min, and 20% A at 60 min. The mobile phases were composed of formic acid in water (1:99 *v*/*v*) as solvent A and acetonitrile as solvent B. The identification of compounds was achieved by comparing the UV absorption spectra and retention times of each compound with those of pure standards injected under the same conditions. The results were expressed as mg/g of flour.

### 3.6. Statistical Assay

Statistical analysis and comparisons among means were carried out using the statistical package SPSS 26.0 (SPSS Inc., Chicago, IL, USA). An ANOVA (one way) was conducted using a confidence level of 95% to determine any significant difference between pitahaya-peel flour and pitahaya-flesh flour. When there was a significant difference (*p* < 0.05), Tukey’s post hoc test was applied to determine the differences among the different pitahaya flours.

## 4. Conclusions

The results obtained suggest that the flours obtained from the peel and pulp of pitahaya (*Hylocereus ocamponis*) constitute potential materials to be utilized as ingredients in the food industry due to their high content of bioactive compounds such as betalains, phenolic acids, and flavonoids, as well as remarkable antioxidant capacity. In addition, these flours showed a considerable content of total dietary fiber, with very interesting techno-functional properties. Given the affordability of pitahaya, it ought to be seen as a promising nutraceutical asset. These resources hold the potential to provide affordable and nutritious dietary supplements for underserved communities. Moreover, they might be harnessed in the pharmaceutical industry as nutritional supplements, leveraging the health benefits associated with dietary fiber and other bioactive compounds.

## Figures and Tables

**Table 1:** Chemical composition of flours obtained from edible and non-edible portions of pitahaya (*Hylocereus ocamponis*) fruit.

	Pitahaya-Peel Flour	Pitahaya-Flesh Flour
Protein ^1^	6.72 ± 0.07 ^a^	6.06 ± 0.19 ^b^
Fat	2.23 ± 0.12 ^a^	2.19 ± 0.07 ^a^
Ash	11.63 ± 0.76 ^a^	3.63 ± 0.01 ^b^
Total Dietary fiber	56.56 ± 0.76 ^a^	8.22 ± 0.76 ^b^
Pectin	24%	1.00%
Calcium	1.64 ± 0.04 ^a^	0.08 ± 0.02 ^b^
Potassium	4.43 ± 0.19 ^a^	0.85 ± 0.05 ^b^
Phosphorus	0.58 ± 0.05 ^a^	0.66 ± 0.03 ^a^

^1^ Values expressed as g/100 g of sample. Values followed by the same letter in the same row indicate that no statistical differences were found (*p* > 0.05) according to Tukey’s HSD post hoc test.

**Table 2:** Physico-chemical properties of flours obtained from edible and non-edible portions of pitahaya (*Hylocereus ocamponis*) fruit.

			Color Coordinates		
	pH	Water Activity	L*	a*	b*
Pitahaya-peel flour	4.83 ± 0.05 ^b^	0.436 ± 0.002 ^b^	55.26 ± 0.69 ^a^	28.48 ± 0.34 ^b^	−1.64 ± 0.08 ^a^
Pitahaya-flesh flour	5.80 ± 0.05 ^a^	0.486 ± 0.004 ^a^	26.12 ± 0.82 ^b^	45.19 ± 0.17 ^a^	−2.10 ± 0.03 ^b^

Values followed by the same letter in the same row indicate that no statistical differences were found (*p* > 0.05) according to Tukey’s HSD post hoc test.

**Table 3:** Techno-functional properties of flours obtained from edible and non-edible portions of pitahaya (*Hylocereus ocamponis*) fruit.

	WHC	OHC	SWC
Pitahaya-peel flour	15.69 ± 0.34 ^a^	4.17 ± 0.52 ^a^	24.80 ± 0.78 ^a^
Pitahaya-flesh flour	1.83 ± 0.38 ^b^	0.31 ± 0.04 ^b^	5.44 ± 0.23 ^b^

WHC: water-holding capacity (values expressed as g water/g sample); OHC: oil-holding capacity (values expressed as g oil/g sample); SWC: swelling capacity (values expressed as mL/g sample). Values followed by the same letter in the same column indicate that no statistical differences were found (*p* > 0.05) according to Tukey’s HSD post hoc test.

**Table 4:** Antioxidant properties of flours obtained from edible and non-edible portions of pitahaya (*Hylocereus ocamponis*) fruit.

	ABTS	DPPH	FIC	FRAP
Pitahaya-peel flour	5.70 ± 0.16 ^a^	1.19 ± 0.05 ^a^	0.09 ± 0.01 ^a^	4.94 ± 0.21 ^a^
Pitahaya-flesh flour	4.73 ± 0.19 ^b^	1.38 ± 0.06 ^b^	0.08 ± 0.01 ^a^	3.12 ± 0.12 ^b^

For ABTS, DPPH, and FRAP, values were expressed as mM Trolox Equivalents/g flour; for FIC, values were expressed as mg EDTA Equivalents/g flour. Values followed by the same letter in the same column indicate that no statistical differences were found (*p* > 0.05) according to Tukey’s HSD post hoc test.

**Table 5:** Polyphenolic profile of flours obtained from edible and non-edible portions of pitahaya (*Hylocereus ocamponis*) fruit.

		Pitahaya-Peel Flour	Pitahaya-Flesh Flour
**Phenolic Acids**	3-*O*-Caffeoylquinic acid	0.28 ± 0.03 ^aG^	0.04 ± 0.03 ^bG^
	4-*O*-Caffeoylquinic acid	0.97 ± 0.05 ^aF^	0.17 ± 0.03 ^bE^
	caffeic acid	0.04 ± 0.00 ^bK^	0.27 ± 0.03 ^aD^
	cichoric acid	n.d.	0.14 ± 0.02 ^EF^
	Sinapic acid	0.14 ± 0.01 ^aJ^	0.11 ± 0.01 ^aF^
	**TOTAL**	**1.38 ± 0.01 ^a^**	**0.79 ± 0.02 ^b^**
**Flavan-3-ol**	Catechin	25.85 ± 0.48 ^aA^	5.32 ± 0.15 ^bA^
	**TOTAL**	**25.85 ± 0.48 ^a^**	**5.32 ± 0.15 ^b^**
**Flavonol**	Quercetin 3-galactoside	4.56 ± 0.13 ^D^	n.d.
	Quercetin 3-rutinoside	3.87 ± 0.12 ^E^	n.d.
	Quercetin 3-*O*-beta-D-glucofuranoside	3.78 ± 0.14 ^aE^	0.04 ± 0.00 ^bJ^
	Quercetin 3-rhamnoside	11.66 ± 0.20 ^aC^	0.07 ± 0.00 ^bH^
	Myricetrin	12.10 ± 0.16 ^aB^	0.08 ± 0.01 ^bH^
	**TOTAL**	**35.97 ± 0.14 ^a^**	**0.19 ± 0.01 ^b^**
**Anthocyanins**	Cyanidin-3-glucoside	0.11 ± 0.01 ^bK^	0.24 ± 0.01 ^aD^
	Cyanidin-3-rutinoside	n.d.	0.32 ± 0.02 ^C^
	Delphinidin-3-glucoside	0.20 ± 0.01 ^bH^	0.34 ± 0.02 ^aC^
	Delphinidin-3-rutinoside	n.d.	0.40 ± 0.02 ^B^
	**TOTAL**	**0.31 ± 0.01 ^b^**	**1.30 ± 0.02 ^a^**
**Anthocyanidins**	Cyanidin	0.13 ± 0.00 ^J^	n.d.
	Delphinidin	0.11 ± 0.01 ^K^	n.d.
	**TOTAL**	**0.24 ± 0.01**	

Values were expressed as mg/g. n.d.: None detected. (mM Trolox Equivalents/g flour); for FIC, values were expressed as (mg EDTA Equivalents/g flour). Values followed by the same small letter in the same row indicate that no statistical differences were found (*p* > 0.05) according to Tukey’s HSD post hoc test. For each flour, values followed by the same capital letter in the same column indicate that no statistical differences were found (*p* > 0.05) according to Tukey’s HSD post hoc test.

## Data Availability

The data presented in this paper are available upon request from the corresponding author.
